# Publisher Correction: Deep-potential enabled multiscale simulation of gallium nitride devices on boron arsenide cooling substrates

**DOI:** 10.1038/s41467-024-47636-3

**Published:** 2024-04-16

**Authors:** Jing Wu, E Zhou, An Huang, Hongbin Zhang, Ming Hu, Guangzhao Qin

**Affiliations:** 1 State Key Laboratory of Advanced Design and Manufacturing Technology for Vehicle, College of Mechanical and Vehicle Engineering, Hunan University, Changsha, 410082 P. R. China; 2 Institut für Materialwissenschaft, Technische Universität Darmstadt, Darmstadt, 64289 Germany; 3 Department of Mechanical Engineering, University of South Carolina, Columbia, SC 29208 USA; 4 Research Institute of Hunan University in Chongqing, Chongqing, 401133 China; 5 Greater Bay Area Institute for Innovation, Hunan University, Guangzhou, 511300 Guangdong China; 6 Key Laboratory of Computational Physical Sciences (Fudan University), Ministry of Education, Shanghai, China; 7 Present Address: School of Energy and Power Engineering, Huazhong University of Science and Technology, Wuhan, 430074 Hubei China

**Keywords:** Composites, Structural properties, Surfaces, interfaces and thin films, Electrical and electronic engineering

Correction to: *Nature Communications* 10.1038/s41467-024-46806-7, published online 25 March 2024

In this article, the author name E Zhou was incorrectly written as E. Zhou. The original article has been corrected.

