# Supplementary material for: Deep-potential enabled multiscale simulation of gallium nitride devices on boron arsenide cooling substrates
Source: Nat Commun. 2024 Mar 25;15:2540. doi: 10.1038/s41467-024-46806-7 (PMC10963741; doi:10.1038/s41467-024-46806-7)
Supplement: Supplementary file 1 — Supplementary Information [file 41467_2024_46806_MOESM1_ESM.pdf]

Supplementary Information

Deep-potential driven multiscale simulation of gallium nitride devices on boron arsenide cooling substrates

Jing Wu[1†], E Zhou[1], An Huang[1], Hongbin Zhang[2], Ming Hu[3], and Guangzhao Qin[1,4,5,6*]

[1]State Key Laboratory of Advanced Design and Manufacturing Technology for Vehicle, College of Mechanical and Vehicle Engineering, Hunan University, Changsha 410082, P. R. China

[2]Institut für Materialwissenschaft, Technische Universität Darmstadt, Darmstadt, 64289, Germany

[3]Department of Mechanical Engineering, University of South Carolina, Columbia, SC 29208, USA

[4]Research Institute of Hunan University in Chongqing, Chongqing 401133, China

[5]Greater Bay Area Institute for Innovation, Hunan University, Guangzhou 511300, Guangdong Province, China

[6]Key Laboratory of Computational Physical Sciences (Fudan University), Ministry of Education

[†] Present address: School of Energy and Power Engineering, Huazhong University of Science and Technology, Wuhan, Hubei 430074, China

[*] Author to whom all correspondence should be addressed. E-Mail: gzqin@hnu.edu.cn

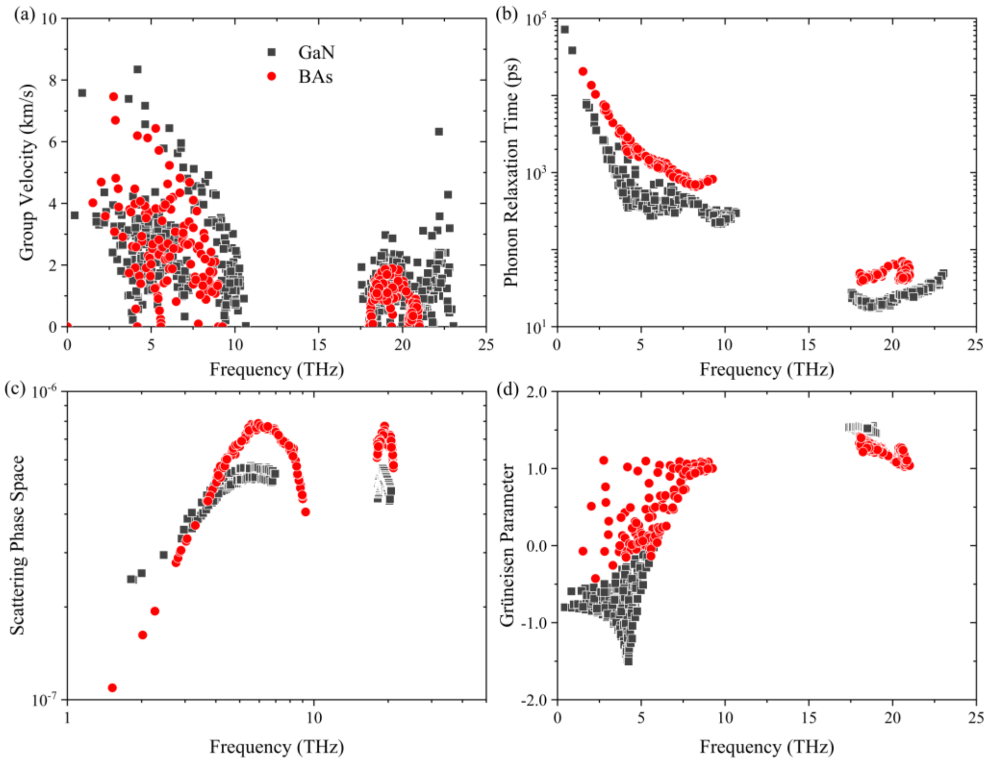

Supplementary Figure 1 The comparison of group velocity, phonon relaxation time, scattering phase space, and Grüneisen parameter between BAs and GaN.

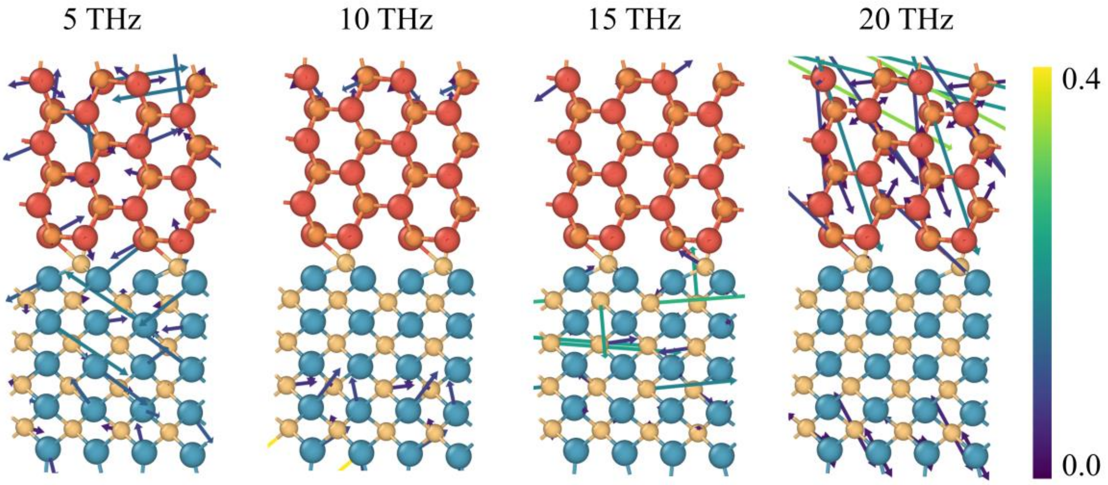

Supplementary Figure 2 The visualization of phonon eigenvectors for GaN-BAs interface at different frequencies. The color bar represents the amplitude of the normalized eigenvectors and the arrow represents the magnitude and direction of the amplitude.

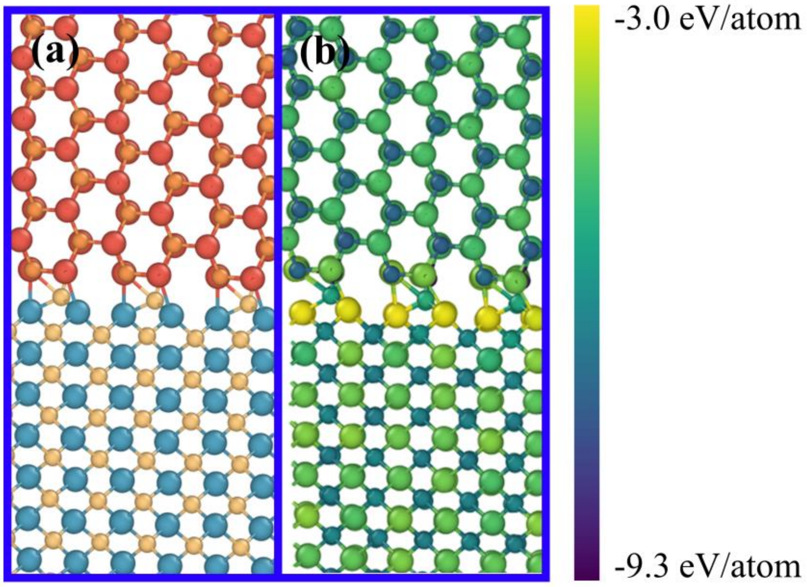

Supplementary Figure 3 (a) GaN-BAs heterostructures after relaxation and (b) the atomic energy distribution. The color bar represents the magnitude of atomic energy.

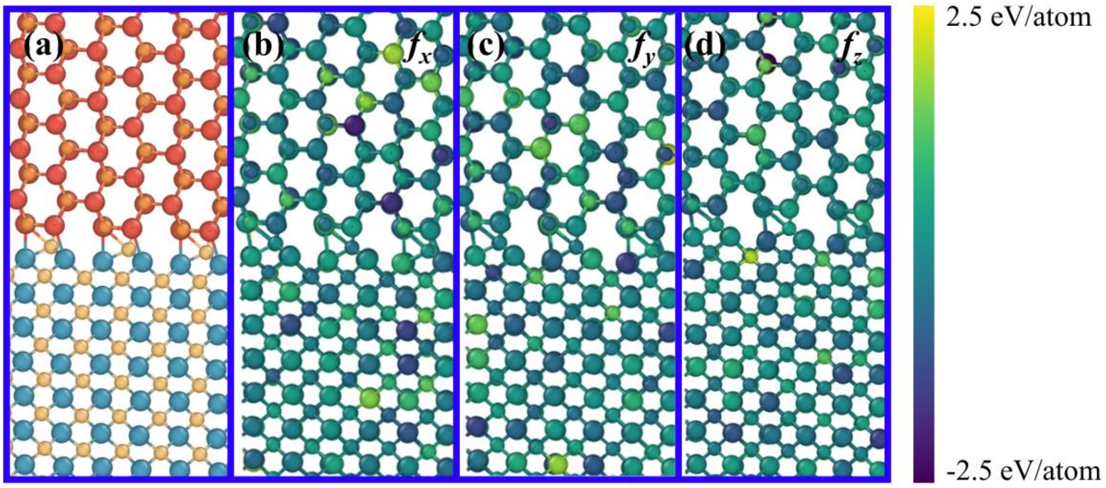

Supplementary Figure 4 Force distribution in GaN-BAs heterostructures. (a) GaN-BAs heterostructure after relaxation. (b) Force f_x in the x-direction of GaN-BAs; (c) Force f_y in the y-direction of GaN-BAs; (d) Force f_z in the z-direction of GaN-BAs. The color bar represents the magnitude of atomic force.

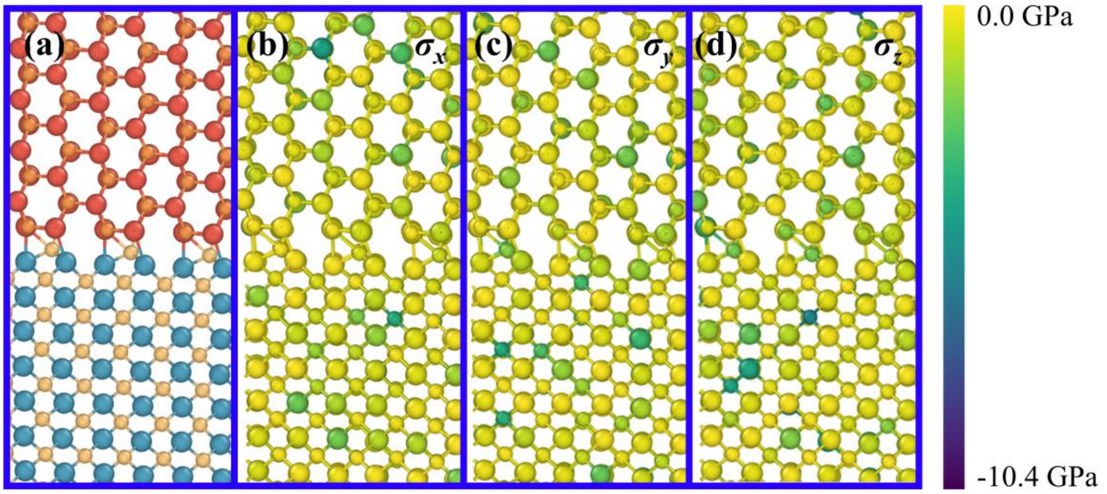

Supplementary Figure 5 Stress distribution in GaN-BAs heterostructures. (a) GaN-BAs heterostructure after relaxation. (b) Stress σ_x in the *x*-direction of GaN-BAs; (c) Stress σ_y in the *y*-direction of GaN-BAs; (d) Stress σ_z in the *z*-direction of GaN-BAs. The color bar represents the magnitude of atomic stress.

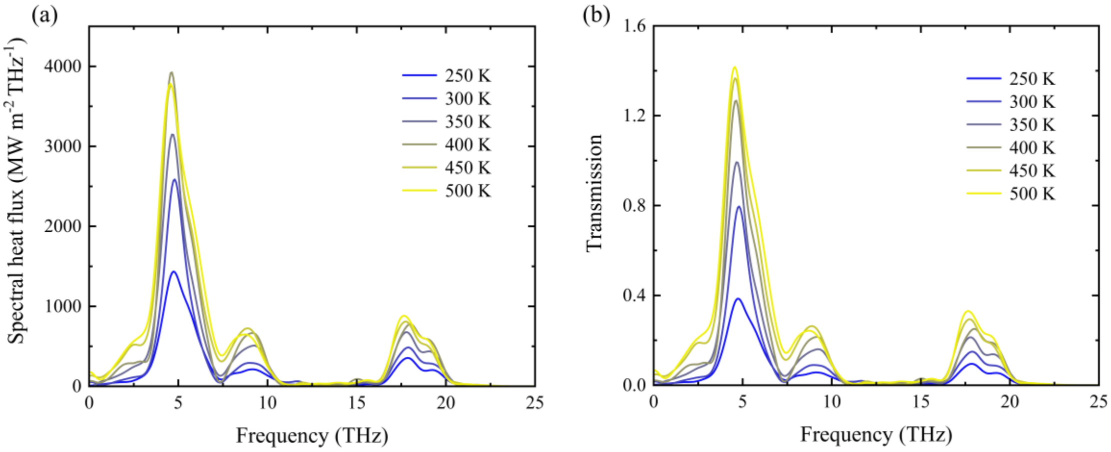

Supplementary Figure 6 The temperature-dependent spectral heat flux and phonon transmission coefficient in GaN-BAs heterostructures.

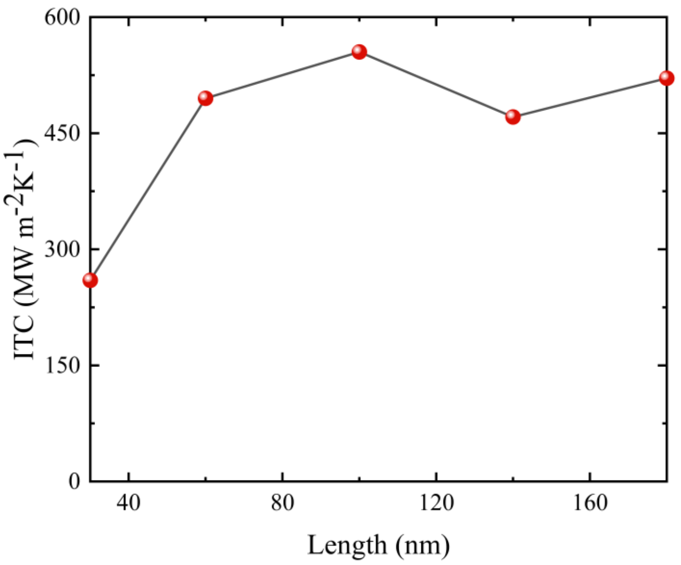

Supplementary Figure 7 The length-dependent ITC in GaN-BAs heterostructures.

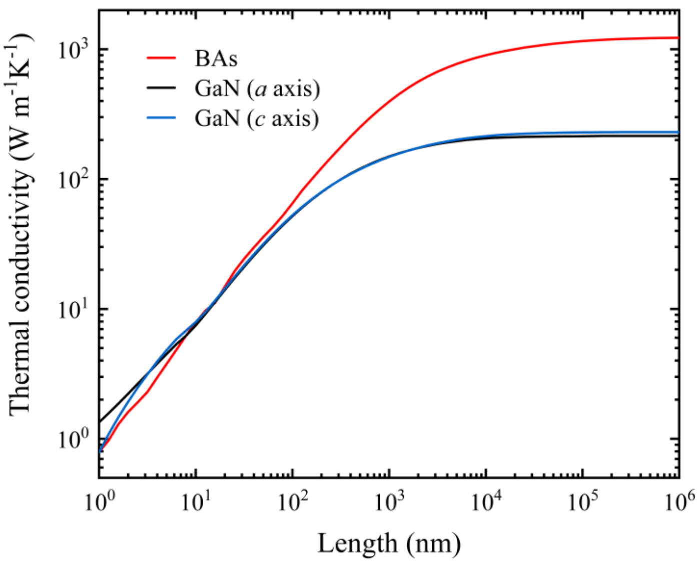

Supplementary Figure 8 The length-dependent thermal conductivity of GaN and BAs.

Supplementary Table. 1 The percentage contribution of κ from the two transverse-acoustic (TA_1 and TA_2), longitudinal-acoustic (LA) and optical branches.

	GaN-inplane	GaN-outplane	BAs-in/outplane
TA_1	21.2	19.7	26.4
TA_2	23.4	27.6	45.1
LA	24.7	21.1	28.4
Optical	30.7	31.6	0.1
